# Baclofen-Induced Encephalopathy in End Stage Renal Disease

**DOI:** 10.1155/2015/203936

**Published:** 2015-07-29

**Authors:** Andrew Meillier, Cara Heller, Shyam Patel

**Affiliations:** Department of Medicine, Temple University Hospital, Philadelphia, PA, USA

## Abstract

Baclofen is a highly used centrally acting GABA agonist that continues to be an effective therapy for spasticity and chronic hiccups. The renally dependent excretion determines the circulating concentrations and guides effective dosing to decrease adverse reactions. Caution should be considered in administering baclofen to patients with decreased renal function. We present a patient with end stage renal disease on hemodialysis with recent baclofen ingestion who presented with toxic encephalopathy that was resolved with additional dialysis sessions.

## 1. Introduction

Baclofen (parachlorophenyl gamma-aminobutyric acid) is an agonist of the neurotransmitter gamma-aminobutyric acid (GABA) [1]. The centrally functioning mechanism of action has been used to treat spasticity and more recently chronic hiccups [2, 3]. Baclofen is well absorbed in the gastrointestinal tract ideally producing the determined therapeutic levels of 80–400 ng/mL [4]. The primary excretion is through the kidney (70–80%) with the remainder metabolized in the liver or processed through the gastrointestinal tract [4]. Only a small portion is able to cross the blood brain barrier, creating the desired effects. The half-life is approximately 6.8 hours [3]. Patients with decreased renal function have an extended half-life with more crossing of the blood brain barrier. The generalized central nervous system (CNS) depression effects are then further pronounced producing fatigue, syncope, hypotension, ataxia, psychological disturbances, and cardiovascular and respiratory depression [2, 4].

In patients with limited renal function, there have been reports of toxic side effects with the initial dosing of 5 mg t.i.d. within only a few days [5]. Baclofen toxicity has been shown to resolve with hemodialysis resembling excretion rates similar to normal renal function [6]. Here we report a case of baclofen-induced encephalopathy in a dialysis patient with recovery following hemodialysis sessions.

## 2. Case Report

Our patient is a 54-year-old male with a past medical history of end stage renal disease on hemodialysis, diabetes mellitus type 2, hypertension, and hepatitis C who presented for altered mental status. The patient had a recent internal medicine clinic visit with concern for pain due to paraspinal muscle spasm. The patient was started on baclofen 10 mg b.i.d. and tramadol 50 mg b.i.d. On the day of admission, the patient was found to be lying on the ground and speaking incoherently by an acquaintance. He was brought to the hospital and admitted to the intensive care unit for altered mental status.

The vital signs were the following: temperature 99.1°F, respiratory rate 21 breaths/minute, pulse 83 beats/minute, blood pressure 138/64 mmhg, and pulse oxygenation 99% on room air. On initial presentation, the patient was lethargic but arousable and unable to answer questions. The remainder of the exam was consistent with his known comorbidities including a left upper extremity arteriovenous fistula and right lower extremity transmetatarsal amputation. Laboratory tests showed the following: sodium 141 mEq/L, potassium 5.2 mEq/L, chloride 101 mEq/L, bicarbonate 26 mEq/L, blood urea nitrogen 55 mg/dL, creatinine 10.98 mg/dL. A white blood cell count was 12.4 mg/dL, hemoglobin 11.3 mg/dL, and platelet count 155/mm^3^. A venous blood gas included a pH of 7.41 and carbon dioxide 44 mmHg. A hepatic function panel had a total bilirubin of 0.8 mg/dL, AST 45 u/L, ALT 26 u/L, alkaline phosphatase 52 u/L, creatine kinase 1738 u/L, and ammonia 52 u/L. A head computed tomography was performed with no acute intracranial abnormalities. An abdominal ultrasound found hepatomegaly with fatty infiltration. A urine toxicology screen was negative. The patient's pharmacy confirmed fulfillment of the baclofen and tramadol prescription 3 days prior to admission. Compliance with his last hemodialysis session was confirmed. With high suspicion for baclofen-induced encephalopathy, the patient was immediately dialyzed. He received hemodialysis for two sessions with significant improvement in mental status without any residual symptoms. Upon discharge, the patient had baclofen discontinued with no similar episodes since the present hospitalization.

## 3. Discussion

Baclofen was originally found to be effective in treatment of spasticity concerning spinal cord lesions in the 1960s [7]. The mechanism of action is a centrally acting presynaptic agonist of GABA to enhance tone reduction and reduce spasticity [1]. In recent studies, it has been shown effective in treating persistent hiccups [3]. Baclofen is efficiently absorbed in the gastrointestinal tract and with variations of the half-life dependent largely on renal function being the primary mechanism of excretion [4]. A high correlation between renal clearance and creatinine clearance was shown to exist in a excretion kinetic study [8].

Baclofen is a moderately lipophilic molecule and with increased circulating concentration crossing the blood brain barrier has a correlating amplified centrally acting response [9]. These adverse reactions include hypotension, bradycardia, respiratory depression, and toxic encephalopathy [5, 9]. There have been previous cases with chronic renal failure and symptoms of neurotoxicity. Serum concentrations of baclofen were measured to have progressively decreased during the sessions of hemodialysis with resolution of the neurologic sequel [5]. The effectiveness of hemodialysis was additionally seen in another case of baclofen overdose with normal renal function. In this case, a patient consumed 200 mg of extended release baclofen with subsequent ventilator dependent respiratory failure. Two sessions resolved the side effects with successful extubation and resolution of additional symptoms [9].

In a study of the efficiency in using hemodialysis, the filtration rate for baclofen was as effective as normal kidneys [6]. This high efficiency of filtration is because only 30% of the circulating concentration is bound to protein [6]. In case reports, there is a several-hour delay prior to improvement in symptoms with the theorized necessity of redistribution from the CNS to the intravascular system and filtration by dialysis [10]. Interestingly, the therapeutic range of baclofen 80–400 ng/mL has been found to cause negative side effects in patients with impaired renal function. The exact therapeutic level with the intended decrease in dosing remains unclear with continued susceptibility to adverse side effects [11].

Electroencephalography (EEG) has been used in initial evaluations for the etiology of unconsciousness in baclofen overdose. These changes include pseudoperiodic sharp waves to periodic high-amplitude discharges and generalized slow waves [12]. Resolution of these EEG changes were seen following treatment [13]. These changes did not indicate the capacity to induce epilepsy and have been recommended not to treat with antiepileptic medications [13].

Baclofen renal dosing continues to be nonspecific with renal adjustment recommendations. A study in 2011 highly recommended changes in labeling with improved guidelines in dosing. There was a proposal to avoid this medication with a GFR < 30 mL/min/1.73 m^2^. This was further detailed with a decrease in dosing and titrating in a GFR > 30 to 60 mL/min/1.73 m^2^ [14]. In following the reported history of decreased renal function and side effects, this continues to be a highly appropriate recommendation. Our case represents toxic encephalopathy with inappropriate dosing of baclofen that was resolved with hemodialysis. Special consideration is required with this renally excreted medication and more specific guidelines would likely further alleviate incidence of side effects.
